# Relating Mutant Genotype to Phenotype via Quantitative Behavior of the NADPH Redox Cycle in Human Erythrocytes

**DOI:** 10.1371/journal.pone.0013031

**Published:** 2010-09-28

**Authors:** Pedro M. B. M. Coelho, Armindo Salvador, Michael A. Savageau

**Affiliations:** 1 Biological Chemistry Group, Chemistry Department, University of Coimbra, Coimbra, Portugal; 2 Biomedical Engineering Department, University of California Davis, Davis, California, United States of America; 3 Center for Neurosciences and Cell Biology, University of Coimbra, Coimbra, Portugal; INSERM U1016, Institut Cochin, France

## Abstract

**Background:**

The NADPH redox cycle plays a key role in antioxidant protection of human erythrocytes. It consists of two enzymes: glucose-6-phosphate dehydrogenase (G6PD) and glutathione reductase. Over 160 G6PD variants have been characterized and associated with several distinct clinical manifestations. However, the mechanistic link between the genotype and the phenotype remains poorly understood.

**Methodology/Principal Findings:**

We address this issue through a novel framework (design space) that integrates information at the genetic, biochemical and clinical levels. Our analysis predicts three qualitatively-distinct phenotypic regions that can be ranked according to fitness. When G6PD variants are analyzed in design space, a correlation is revealed between the phenotypic region and the clinical manifestation: the best region with normal physiology, the second best region with a pathology, and the worst region with a potential lethality. We also show that *Plasmodium falciparum*, by induction of its own *G6PD* gene in G6PD-deficient erythrocytes, moves the operation of the cycle to a region of the design space that yields robust performance.

**Conclusions/Significance:**

In conclusion, the design space for the NADPH redox cycle, which includes relationships among genotype, phenotype and environment, illuminates the function, design and fitness of the cycle, and its phenotypic regions correlate with the organism's clinical status.

## Introduction

The NADPH redox cycle plays a key role in the oxidative stress response of human erythrocytes. It consists of two enzymes: glucose-6-phosphate dehydrogenase (G6PD, EC 1.1.1.49) and glutathione reductase (GSR, EC 1.8.1.7). Although variants of G6PD have been intensively studied and are associated with several distinct clinical manifestations, the relationship between the genotype and the phenotype is still poorly understood. To address this issue, we have constructed a “system design space” which facilitates the quantitative comparison of wild-type and variants for the redox cycle. Our results identify three different phenotypes that correlate with clinical manifestations.

**Figure 1:**
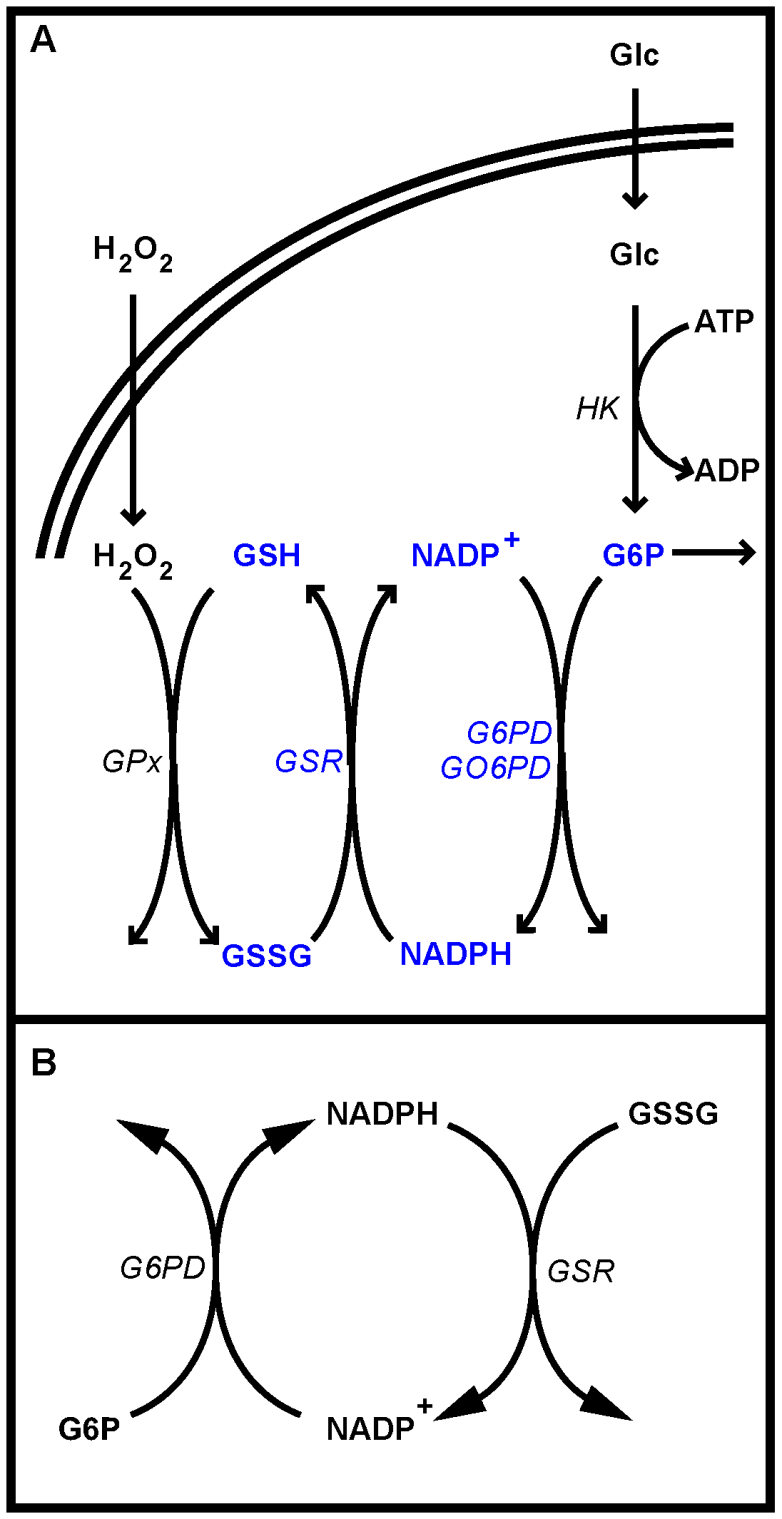
Oxidative part of the hexose monophosphate shunt and core reactions of the NADPH redox cycle. Schematic representations of (A) the shunt and relevant sources of oxidative load and (B) the NADPH redox cycle. Abbreviations: ADP – adenosine 5-diphosphate, ATP – adenosine 5-triphosphate, Glc – glucose, G6P – glucose 6-phosphate, *G6PD* – glucose 6-phosphate dehydrogenase, *GO6PD* – gluconate 6-phosphate dehydrogenase, *GPx* – glutathione peroxidase, GSH – reduced glutathione, *GSR* – glutathione reductase, GSSG – oxidized glutathione, *HK* – hexokinase, NADP^+^ – oxidized nicotinamide adenine dinucleotide phosphate, NADPH – reduced nicotinamide adenine dinucleotide phosphate.

G6PD catalyses the first step of the hexose-monophosphate shunt (**Figure 1A**), which provides pentoses for nucleic acid synthesis and regenerates NADPH. In erythrocytes, NADPH is required for various processes, but most of it is oxidized by GSR [1]. The latter process regenerates reduced glutathione (GSH) that is oxidized in the repair of oxidative damage. In mice, and presumably in other organisms, G6PD is dispensable for pentose synthesis but essential for defense against oxidative stress [2]. High levels of G6PD exist for this function, but under pronounced oxidative stress hexokinase (EC 2.7.1.1) becomes rate-limiting for the NADPH supply [3].

Previous quantitative analysis of the NADPH redox cycle [4], [5] indicates that normal G6PD activity is sufficient but not superfluous to avoid NADPH depletion and ensure timely adaptation of the NADPH supply during pulses of oxidative load such as those that occur during adherence of erythrocytes to phagocytes. The quantitative analysis of this system has been facilitated by two recent developments: a method for constructing the “system design space” [6] and a related method for calculating “global tolerances” [7] to large variations in the values of system parameters and environmental inputs.

In this paper we utilize the design space as a framework to compare the quantitative phenotypes of wild-type and mutant variants of the NADPH redox cycle. In particular, 160 G6PD variants have been characterized [8], and there are several distinct phenotypes associated with G6PD deficiency [9]. This system presents a unique opportunity to relate genotype to phenotype by focusing on the quantitative behavior of the integrated NADPH redox cycle. Our analysis of this system and its mutants requires additional background regarding its biochemistry, genetics and clinical manifestations.

G6PD in its active form is made up of two or four identical subunits, each with a molecular mass of 59 kDa. The gene for G6PD is on the X-chromosome, and the deficiency is inherited in a sex-linked fashion. Hemizygous males and homozygous females with low-activity mutant forms of the enzyme carry only G6PD-deficient erythrocytes. However, female heterozygotes carry both normal and deficient erythrocytes. The latter outcome is due to the fact that each cell inactivates one of its X-chromosomes, chosen at random. The most common G6PD form worldwide is the B form. However, in Africa up to 40% of the population carry the non-deficient A form of G6PD [10]. The most common G6PD-deficient variant in Africa is the A- allele with a frequency between 0 and 25% [11].

Over 400 million people in the world suffer from G6PD deficiency; which makes it the most common known enzymopathy. The highest prevalence rates are found in tropical and subtropical regions of the world and in some areas of the Mediterranean. It would be difficult to understand the existence of such a widespread enzymatic deficiency without a counterbalancing biological advantage. The observation that the G6PD A- mutation has fitness costs such as making individuals more prone to develop hemolytic anemias [12] makes this issue even more intriguing. In 1960, Allison [13] and Motulsky [14] first suggested that individuals deficient in G6PD might be at a selective advantage in malaria endemic areas. However, numerous clinical studies have yielded conflicting results over the alleged protective role of G6PD deficiency against malaria. In particular, clinical studies of severe malaria have shown a protective role for G6PD deficiency in hemizygous males [15], [16] while, in heterozygous females, there is either a protective effect [16] or no effect [15]. The studies relating to uncomplicated malaria have shown more conflicting results with heterozygous females protected [16], [17], [18], [19], at increased risk [20], or unaffected by G6PD deficiency [21]. In a recent paper, *Johnson M.K. et al.* [22] compared the association between uncomplicated malaria incidence and G6PD deficiency using the two different methods currently available: G6PD enzyme activity and G6PD genotype assessment. They found a 52% reduced risk of uncomplicated malaria in G6PD-deficient females when the deficiency was assessed using enzyme activity. When the assessment method was based on the genotype, the protective association was no longer significant, which is likely due to the large dispersion in enzymatic activity caused by random X-chromosome inactivation [22]. For G6PD deficient males, there was no association with the incidence of uncomplicated malaria regardless of assessment method. Their work suggests that the conflict between prior association studies might be related to the method of G6PD assessment.

G6PD variants have been described on the basis of their biochemical properties and grouped into five classes according to the level of enzyme activity and clinical manifestations. In 1967, a committee of the World Health Organization [23] recommended standard techniques for the biochemical characterization of G6PD variants. Class I includes severely deficient variants that suffer from chronic non-spherocytic haemolytic anemia. Class II variants have less than 10% enzyme activity but do not suffer from chronic non-spherocytic haemolytic anemia. Class III variants are moderately deficient (10–60% activity). Class IV has normal enzyme activity (60–150%) and in Class V the enzyme activity is higher than normal (>150% activity) [24]. Of the 400 million people worldwide that exhibit G6PD deficiency, there are, for instance, 4 million African Americans (1 in 10) [25] in a country where malaria has been eradicated since 1951 [26]. Thus, the implications of G6PD deficiencies unrelated to malaria represent a significant medical problem [27].

In terms of clinical manifestations, G6PD deficiency can be divided into 3 groups. The first group (Class IV and V) consists of variants that do not have any clinical disorder. The second group (Class II and III) shows a hemolytic reaction that is triggered by certain drugs, by infection or by the ingestion of certain foods. The mechanism of hemolysis is not known, but it has been proposed that it results from the inability of G6PD-deficient erythrocytes to cope with the oxidative damage produced by the agents mentioned above [28]. The third group (Class I) may be classified as having chronic non-spherocytic hemolytic anemia, which is exacerbated by oxidative stress.

In this paper, we assembled existing data into an integrated model of the NADPH redox cycle that allowed us to address the following questions: given the variability in G6PD, how do mutations affect the quantitative performance of the cycle? Is there a correlation between clinical manifestations and poor performance of the NADPH redox cycle? Finally, how does infection of erythrocytes by *Plasmodium falciparum* affect the redox cycle. We base our analysis of these questions on the model shown in **Figure 1B**.

## Methods

### Model Formulation

Under physiological conditions, the steps catalyzed by G6PD and GSR are essentially irreversible [29]. The kinetic parameters of each enzyme and the nominal concentrations of their reactants have been well characterized (**Table 1**).

**Table 1:** Values of the kinetic parameters and concentration variables for the enzymes of the NADPH redox cycle.

Enzyme	Parameter	Value	Ref.
G6PD (B-form)	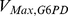	*130 µM s^−1^	[40]
	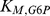	38 µM	[41]
	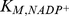	6.5 µM	[41]
	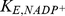	7.9 µM	[41]
	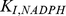	7.1 µM	[41]
	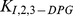	2.3 mM	[3]
	[G6P]	39 µM	[42]
	[2,3-DPG]	3.1 mM	[29]
GSR	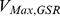	49 µM s^−1^	[40]
	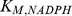	8.5 µM	[43]
	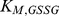	65 µM	[43]
	[GSSG]	0.16 µM	[4]
	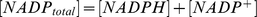	2.7 µM	[4]

*The maximum velocity for synthesizing NADPH is twice the maximum velocity of G6PD (to take into account the GO6PD activity). For further details see [4].

#### G6PD Kinetics

The rate expression for G6PD follows a compulsory order mechanism in which 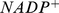 binds first to the enzyme:
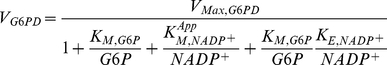
(1)here,
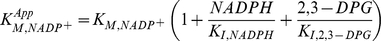
is the apparent Michaelis-Menten constant for NADP^+^ after taking into account the competitive inhibition by NADPH and 2,3-diphosphoglycerate (2,3-DPG). In **Table 1**, we present the experimental values of kinetic parameters for G6PD encoded by the B-form of the gene.

#### GSR Kinetics

At concentrations of oxidized glutathione (GSSG) typical of low to moderate oxidative loads, GSR follows a ping-pong mechanism with rate expression:
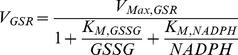
(2)The kinetic parameters of GSR are given in **Table 1**.

### Piecewise Power-Law Representation

Using the method outlined in [7], we combined the fundamental enzyme kinetic rate laws and the conservation of the NADP moiety into a system of ordinary differential equations representing mass balance, which can then be solved to determine the steady-state of integrated system. With this approach, we are able to formulate the piecewise power-law representation of G6PD [Eq(3)] and GSR [Eq (4)] kinetics in normalized form:
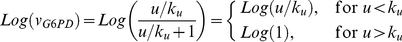
(3)and
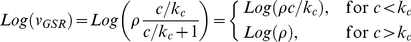
(4)where:
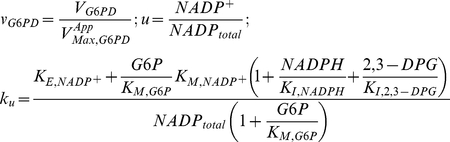


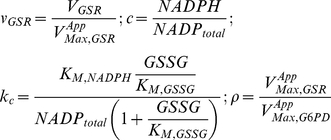


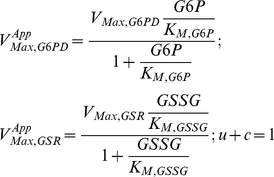
Note that 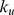 represents the apparent Michaelis constant for NADP^+^ scaled by NADP_total_ and it takes into account product-inhibition and the inhibition by 2,3-DPG. Conversely, 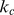 represents the apparent Michaelis constant for NADPH scaled by NADP_total_. The parameter ρ represents the ratio between the apparent maximal velocity of GSR and the apparent maximal velocity of G6PD.

**Figure 2:**
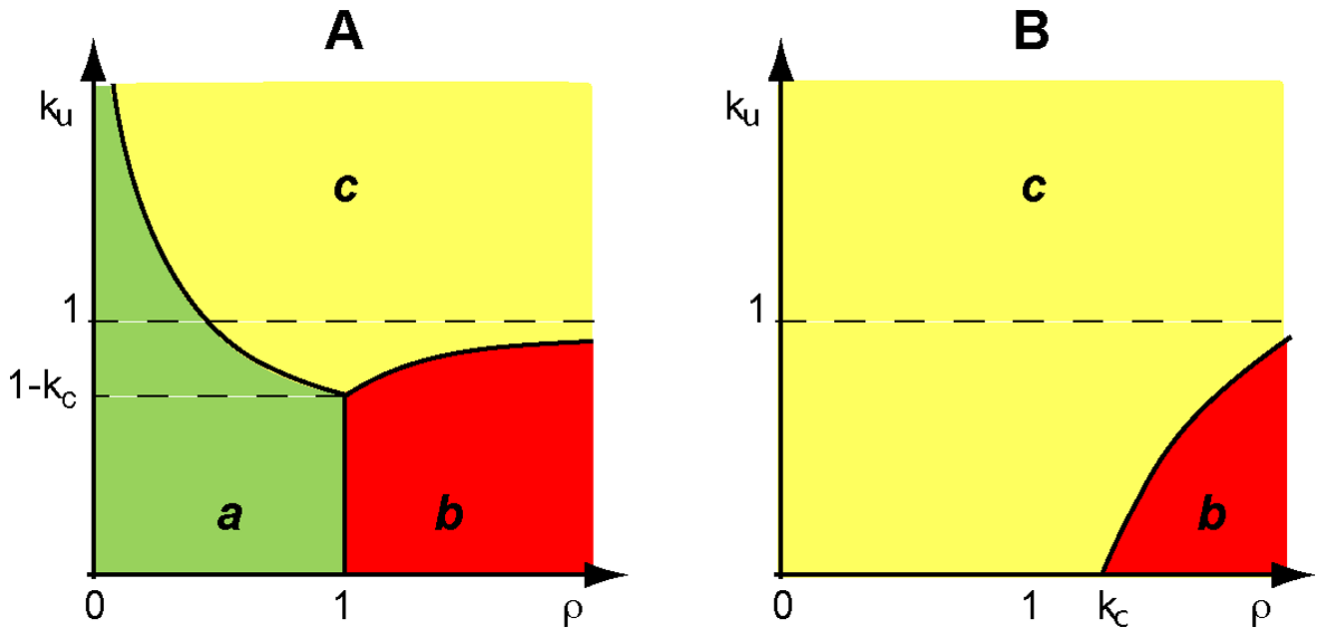
Design space of the NADPH redox cycle. Three distinct operating regimes labeled ***a***, ***b*** and ***c*** are depicted for the cycle in **Figure 1B** (see **Table 2**). In Panel **A**, the apparent Michaelis constant for NADPH is smaller than the total concentration of NADP which implies 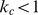 (see Eqns. 3 and 4). In Panel **B**, the relationship is reversed and, therefore, 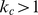. The x-axis, rho, represents the ratio between the apparent maximal velocity of GSR and the apparent maximal velocity of G6PD. The y-axis, 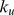 represents a scaled apparent Michaelis constant for NADP^+^ (see Eqns. 3 and 4).

Based on this representation, we recognize that the NADPH redox cycle in human erythrocytes can operate under three different meaningful steady-state regimes (**Table 2**) and one unrealistic case (with 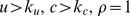) (see Text S1). Each systemic regime will only apply to a particular region of the design space (see **Figure 2**), which is obtained by using the methods previously described [6], [7].

**Table 2:** Steady-state values for fluxes and concentrations of the NADPH redox cycle in each regime.

Regime	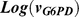	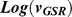	Steady-State Equation*
***a***	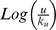	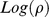	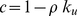
***b***	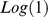	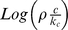	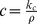
***c***	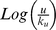	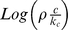	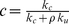

*Note that 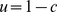 and that 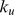 involves the dependent variable 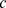. The normalized symbols in this table can be expressed in terms of the original parameters by means of equations 3 and 4. For a complete description of the steady-state solutions see Text S1.

### Determination of System Behavior within each Regime

The system representation within each regime is a simple but nonlinear S-system equation for which determination of local behavior, after appropriate transformation, reduces to conventional linear analysis [30]. Thus, the local behavior is completely determined and readily characterized by the evaluation of the following quantitative indices.

#### Logarithmic gains

The change of concentration (*e.g.*, 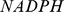) or flux in response to a change in value for an independent variable (*e.g.*, 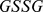) is defined by a relative derivative of the explicit steady-state solution. For example,
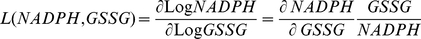
(5)


#### Parameter sensitivities

The change of concentration or flux in response to a change in value for one of the parameters that define the structure of the system (*e.g.*, Michaelis constants) is also determined by a relative derivative of the explicit steady-state solution. For example,
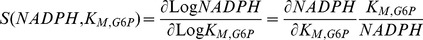
(6)


#### Response time

The eigenvalue, which in the present case is an inverse measure of the system's response time, is determined by a first-order Taylor series approximation in logarithmic space of the differential equation about the steady-state that applies for each systemic regime.

### Criteria for Functional Effectiveness

The performance of the NADPH redox cycle can be evaluated in each systemic regime according to the following quantitative criteria:

The concentration of 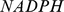 should be well buffered against:

Criterion *1*: fluctuations in the values for the kinetic parameters of G6PD and GSR and of the total amount of NADP present in the erythrocyte;
Criterion *2*: changes in the concentration of 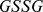;
Criterion *3*: changes in the concentration of 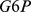.

The supply of 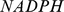 (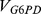) should

Criterion *4*: be responsive to changes in the concentration of 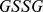.

The sensitivity of the supply of 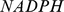 to changes in the concentration of 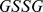 should

Criterion *5*: be well buffered against fluctuations in the values for the kinetic parameters of G6PD and GSR and of the independent variables;

The response time should

Criterion *6*: be fast, and
Criterion *7*: well buffered against fluctuation in the values for the kinetic parameters and independent variables.

### Statistical Tests

All of the statistical tests described below, except one, were performed with algorithms in Mathematica available through reference [31].

#### Parametric

Single-factor Analysis of Variance (ANOVA) is a method used to decide whether differences exist among several population means. It requires all populations to be normally distributed (D'Agostino-Pearson Test for nonnormality) and exhibit the same variance (Bartlett's test for equality of variance). We tried to transform the data so as to satisfy these assumptions whenever either was violated. Whenever the ANOVA rejected equality of all means we investigated each pairwise comparison of population means for significant differences using Tukey's honestly significant difference (HSD) test.

#### Non-Parametric

Whenever the transformation procedure failed to satisfy the ANOVA assumptions, we used a nonparametric method: the Kruskal-Wallis One Way Analysis of Variance. If it was significant, it indicated that at least one of the populations was different from at least one of the others. We used a procedure called “kruskalmc” written for R (included in package pgirmess [32]) to perform a multiple comparison test between populations after the Kruskal-Wallis test (the nonparametric equivalent of a Tukey's honestly significant difference test). This test helped to determine which populations were different in pairwise comparisons adjusted appropriately [33].

## Results

The local performance within the three systemic regimes is determined by the above methods and evaluated according to the previously defined criteria. Our aim is to ascertain which of the systemic regimes is better suited for effective performance of the NADPH redox cycle.

### Analysis of Local Performance

In **Table 3**, we summarize the results from the analysis of local performance in Systemic Regimes ***a***, ***b*** and ***c*** (for further details see Text S1). These results show that local performance in Systemic Regime ***a*** fulfills all the criteria defined above. In contrast, performance in Systemic Regimes ***b*** and ***c*** cannot fulfill criteria *4* and *5* because there is no response to changes in the concentration of GSSG. Moreover, the local robustness (criterion *1*) of systems represented in Systemic Region ***b*** is significantly worse than that of systems in either Systemic Region ***a*** or ***c*** (see Text S1).

**Table 3:** Evaluation of the local performance in Systemic Regimes ***a***, ***b*** and ***c***.

	Capable of being fulfilled by regime:		
Criterion*	*a*	*b*	*c*
*1*	+	+	+
*2*	+	+	+
*3*	+	+	+
*4*	+	−	−
*5*	+	−	−
*6*	+	+	+
*7*	+	+	+

*The criteria were listed under the sub-section “Criteria for Functional Effectiveness” of the Methods. The symbol “+” indicates been able to fulfill the criterion by a particular regime while the symbol “−” indicates the failure to satisfy a given criterion.

Additionally, although Systemic Regimes ***b*** and ***c*** can exhibit a fast response time (Criterion *6*), it will not be with respect to changes in GSSG. Therefore, the importance of this responsiveness is questionable.

In summary, we predict that, under basal conditions, the NADPH redox cycle should operate in Systemic Regime ***a***, which has the best overall local performance. Moreover, natural selection should maintain the operating point far from the boundary to region ***c***, which has less desirable overall local performance, and especially from the boundary to region ***b***, which has the worst performance.

### Analysis of Global Tolerance

The boundaries of Systemic Regime ***a*** are obtained by inserting the linear solution into the corresponding linear breakpoint conditions:
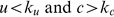
(7)The result is the following set of boundaries
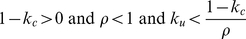
(8)Equations (8) can be represented in terms of the fundamental parameters and variables by substituting the definitions given previously
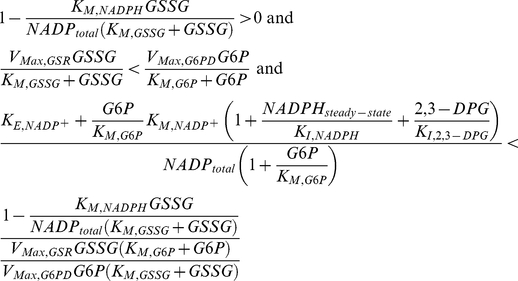
(9)in which:
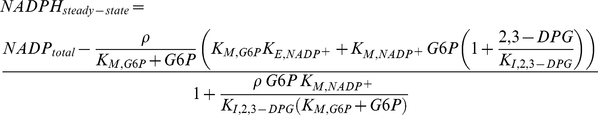
Systems represented within these boundaries exhibit the best local performance and thus these boundaries provide the basis for the analysis of global tolerance.

### Analysis of the normal G6PD (B form)

**Figure 3:**
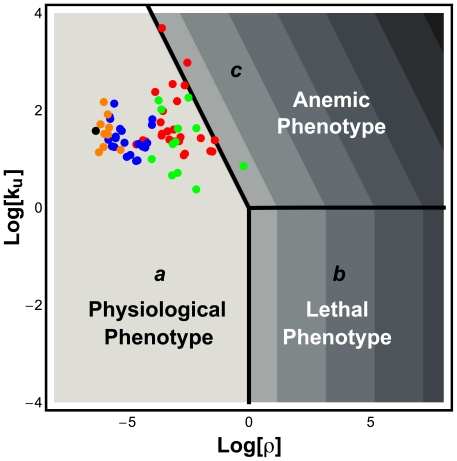
Design space of the NADPH redox cycle and location of G6PD variants. The shading — (white) High to (black) Low — indicates the logarithm of the normalized steady-state concentration of NADPH, 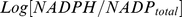. The Log-Log coordinates provide a more convenient representation of the design space that was shown with Cartesian coordinates in **Figure 2A**. The three Systemic Regions are denoted ***a*** (physiological phenotype), ***b*** (potentially lethal phenotype), and ***c*** (pathological phenotype associated with anemia). Symbols: black - normal operating point (G6PD-B form); orange - Class IV variants of G6PD; blue - Class III variants of G6PD; green - Class II variants of G6PD; red - Class I variants of G6PD. Further details are described in the legend of **Figure 2**.

The most common variant of G6PD worldwide is the B form. Given the detailed description of this variant form of G6PD (**Table 1**) and of GSR, we are able to locate the operating point for G6PD B (**Figure 3**, black circle) in the design space and show the normalized steady-state concentration of NADPH in the z-direction with a heat map.

The local behavior of the NADPH redox cycle can be evaluated according to the seven criteria (**Table 4**, **upper panel**) described earlier and, given the boundaries surrounding Systemic Region ***a*** (Eq. 9), we are able to determine the numerical value of global tolerance for each of the kinetic parameters and independent concentration variables (**Table 4**, **lower panel**). We will use the expression “
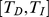
” to describe the global tolerances, where 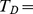
tolerance to a fold decrease and 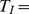
tolerance to a fold increase (since boundaries can be crossed either by decreasing or increasing a parameter).

**Table 4:** Evaluation of Local Performance and Global Tolerance for the NADPH redox cycle in human erythrocytes with the B-form (normal) of G6PD.

Local Performance			
Criterion§	Preference|	Quantitative value†	Optimum value‡
*1*	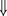	1.05	1
*2*	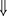	0.01	0
*3*	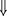	0.00	0
*4*	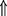	1.00	1
*5*	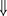	0.00	0
*6*	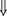	0.20	0
*7*	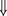	3.49	2

§The criteria were listed under the sub-section “Criteria for Functional Effectiveness” of the Methods Section;

|To improve performance, one must have either a high (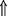) or a low (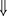) value for the associated criterion;

†The quantitative value was determined by using the data from **Table 1** and the analytical expressions listed in **Table S1, S2** of the **Text S1**;

‡The optimum value was determined analytically except for criterion 7 which had to be determined numerically;

*[T represents tolerance to a fold decrease and T] represents tolerance to a fold increase.

As can be seen from the results in **Table 4** (**upper panel**), natural selection results in a design that has nearly optimal local performance when we consider the normal variant of G6PD (B-form). In a similar trend, the global tolerances (**Table 4**, **lower panel**) range from the smallest of 63 fold to the largest of 1.4×10^5^. The smallest value is associated with the concentrations of 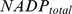 and 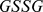, whereas the largest is associated with the product inhibition constant of G6PD.

### Analysis of the G6PD variants

The biochemical properties of more than 400 putative variants have been tabulated in a review [9]. Detailed comparisons of the tabulated values and the values in the corresponding original literature show a number of irregularities. Thus, we have systematically re-evaluated the original literature and produced a revised set of tables for the various classes that highlights the discrepancies (see Text S2).

After eliminating the anomalous cases, we focused on the remaining G6PD variants for which there are documented experimental values for: 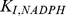, 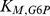, 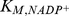 and 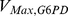. Furthermore, all variants under analysis were characterized according to the standard techniques that the World Health Organization [23] recommended in 1967. We excluded variants (see [9] and Text S2) for which one or more of the kinetic parameters is unknown, or whose kinetics were characterized according to non-standard techniques.

In order to analyze the local performance and global tolerance of the NADPH redox cycle in which variants of G6PD participate, we assumed that 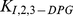, 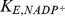, the kinetic parameters of GSR and all the independent variables retain their normal value.

#### Location of the G6PD Variants in Design Space

In **Figure 3**, we display the location of the operating point of the G6PD variants in the Design Space for the NADPH redox cycle. Class III (blue circle) and Class IV (orange circle) variants have their operating points located well within Systemic Region ***a***. Moreover, we can visually ascertain that Class IV variants are further away from Systemic Region ***c*** than Class III variants. However, Class II (green circle) and Class I (red circle) variants have operating points that span Systemic Regions ***a*** and ***c***. This result proves important because we know that the Class I and II variants have clinical manifestations. Therefore, there is a correlation between a Class being near to or present in Systemic Region ***c*** and the existence of clinical problems. Furthermore, several considerations suggest that if the operating point of a variant of G6PD were to be located within Systemic Region ***b***, this might be lethal to the host. First, Systemic Region ***b*** has the worst local performance. Since being present in Systemic Region ***c*** is already associated with life-threatening medical conditions, we can only expected that being present in an even worse phenotypic region could be lethal. Second, no known variant of G6PD leads to an operating point located within Systemic Region ***b***. Moreover, the observation that all known variants of G6PD are located in the design space (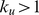) such that an increase in oxidative stress would never move their operating points into Systemic Region ***b*** suggests a selective pressure to prevent even temporary excursions into this region.

#### Local Performance of G6PD Variants

The local behavior of the NADPH redox cycle can be evaluated according to the seven criteria described in the **METHODS** section and our aim is to distinguish among the several classes of G6PD variants (**Table 5**). Note that we are only considering the G6PD variants that lead to systems whose operating points lie within Systemic Region ***a***. Moreover, in this Regime the fulfillment of Criteria *4* and *5* only depends on the concentration of GSSG and on the Michaelis constant of GSR with respect to GSSG (see Text S1), which are fixed in our analysis. Thus, our comparisons will be limited to criteria *1*, *2*, *3*, *6* and *7*.

**Table 5:** Statistical comparison of the local performance of NADPH redox cycles that harbor G6PD variants that belong to different classes.

Class		Criterion§				
Reference	Comparison	*1* *	*2*	*3*	*6*	*7*
I		16.56	2.56	0.96	6.58	3.56
		±52.80	±8.66	±3.39	±5.08	±0.18
	II	*4.77* †	*0.62*	*0.18*	*5.54*	*3.54*
		*±6.42*	*±1.05*	*±0.24*	*±4.66*	*±0.19*
	III	**1.22** ‡	**0.04**	**0.01**	**0.76**	*3.50*
		**±0.19**	**±0.03**	**±0.01**	**±0.60**	*±0.10*
	IV	**1.09**	**0.01**	**0.01**	**0.32**	*3.50*
		**±0.03**	**±0.01**	**±0.00**	**±0.11**	*±0.14*
II		4.77	0.62	0.18	5.54	3.54
		±6.42	±1.05	±0.24	±4.66	±0.19
	III	**1.22**	**0.04**	**0.01**	**0.76**	*3.50*
		**±0.19**	**±0.03**	**±0.01**	**±0.60**	*±0.10*
	IV	**1.09**	**0.01**	**0.01**	**0.32**	*3.50*
		**±0.03**	**±0.01**	**±0.00**	**±0.11**	*±0.14*
III		1.22	0.04	0.01	0.76	3.50
		±0.19	±0.03	±0.01	±0.60	±0.10
	IV	*1.09*	**0.01**	**0.01**	**0.32**	*3.50*
		*±0.03*	**±0.01**	**±0.00**	**±0.11**	*±0.14*

The class in the first column (Reference) has its mean local performance compared to that of the classes in the second column (Comparison). To improve local performance, one must have low values for the criteria.

§The criteria were listed under the sub-section “Criteria for Functional Effectiveness” of the Methods Section.

*Parametric methods of analysis were used in all cases except for criterion 1, in which case the analysis was conducted using non-parametric methods that are based on the comparison of medians. In this case the medians are: 2.65, 2.39, 1.16 and 1.08 for Classes I through IV, respectively.

†Values italicized: p-value>0.05 (insignificant);

‡Values bold: p-value<0.05 (significant).

From the results in **Table 5**, we conclude that the worst local performance is given by systems for which G6PD variants are in Class I or II. In detail, according to criteria *1*, *2*, *3* and *6*, the local performance of systems in Class I or II is statistically worse than those in either Class III or IV. Furthermore, there is no criterion that allows us to distinguish between Class I and Class II, whereas the local performance of a cycle with a Class IV variant is statistically better than a cycle with a Class III variant according to criteria *2*, *3* and *6*.

#### Global Tolerance of G6PD Variants

Once again, we are only considering the G6PD variants that lead to systems operating within Systemic Region ***a***. Therefore, we will determine how much a given kinetic parameter or independent concentration variable has to change in order to place the NADPH redox cycle in a different Systemic Region (**Table 6**).

**Table 6:** Statistical comparison of the global tolerances of NADPH redox cycles harboring G6PD variants that belong to different classes.

Class		Global Tolerance§							
Reference	Comparison	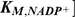	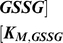	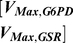	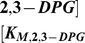	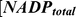	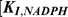 (10^3^)	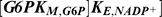	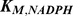 ]*
I		10.8	6.3	6.8	18.1	6.2	30.4	20.1	86.2
		±13.7	±6.0	±7.0	±23.9	±5.8	±73.4	±23.2	±34.5
	II	*18.0* †	*6.5*	*6.8*	*30.7*	*6.4*	*112.0*	*16.0*	*94.4*
		*±22.2*	*±4.8*	*±5.3*	*±38.8*	*±4.6*	*±217.7*	*±11.6*	*±29.2*
	III	**71.8** ‡	**33.3**	**42.7**	**124.4**	**30.8**	**220.7**	**126.6**	**123.0**
		**±37.9**	**±13.7**	**±20.9**	**±66.0**	**±11.9**	**±216.1**	**±81.1**	**±3.5**
	IV	**149.3**	**54.7**	**81.9**	**259.5**	**48.4**	**1407.0**	**236.0**	**125.5**
		**±89.5**	**±15.0**	**±34.2**	**±155.9**	**±11.5**	**±2663.7**	**±94.7**	**±0.7**
II		18.0	6.5	6.8	30.7	6.4	112.0	16.0	94.4
		±22.2	±4.8	±5.3	±38.8	±4.6	±217.7	±11.6	±29.2
	III	**71.8**	**33.3**	**42.7**	**124.4**	**30.8**	*220.7*	**126.6**	**123.0**
		**±37.9**	**±13.7**	**±20.9**	**±66.0**	**±11.9**	*±216.1*	**±81.1**	**±3.5**
	IV	**149.3**	**54.7**	**81.9**	**259.5**	**48.4**	**1407.0**	**236.0**	**125.5**
		**±89.5**	**±15.0**	**±34.2**	**±155.9**	**±11.5**	**±2663.7**	**±94.7**	**±0.7**
III		71.8	33.3	42.7	124.4	30.8	220.7	126.6	123.0
		±37.9	±13.7	±20.9	±66.0	±11.9	±216.1	±81.1	±3.5
	IV	**149.3**	**54.7**	**81.9**	**259.5**	**48.4**	*1407.0*	**236.0**	*125.5*
		**±89.5**	**±15.0**	**±34.2**	**±155.9**	**±11.5**	*±2663.7*	**±94.7**	*±0.7*

The class in the first column (Reference) has its mean Global Tolerance compared to those of the classes on the second column (Comparison). To improve global tolerance, one must have high values.

*Parametric methods of analysis were used in all cases except for the global tolerance of *K_M,NADPH_*, in which case the analysis was conducted using non-parametric methods that are based on the comparison of medians. In this case the medians are: 99.6, 103.2, 124.1 and 125.6 for Classes I through IV, respectively.

†Values italicized: p-value>0.05 (insignificant);

‡Values bold: p-value<0.05 (significant.

§[T represents tolerance to a fold decrease and T] represents tolerance to a fold increase.

From the analysis of **Table 6**, we find that the worst global tolerances are associated with systems of Class I or II. According to all the kinetic parameters and independent variables except 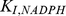, the global tolerances of Class I or II cycles are statistically worse than those of either Class III or IV. In addition, we see that there is no global tolerance that allows us to distinguish between Class I and Class II. Furthermore, the analysis of global tolerance, except for 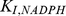 and 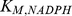, allows us to differentiate between Class III and Class IV and conclude that Class IV has better global tolerances than Class III.

#### Distinguishing Class I from Class II Variants

Our analysis has shown that there is no local performance criterion or global tolerance that distinguishes a NADPH redox cycle with a Class I variant from one with a Class II variant of G6PD. However, the clinical manifestations are quite different. How might these differences be reconciled? One possibility is differences in kinetic properties of Class I and Class II variants of G6PD. In **Table 7**, we display the medians of the kinetic properties of the G6PD variants, when grouped into their respective classes. These results show that it is possible to make this distinction on the basis of 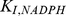: the median of Class I is 7.6 µM while the median of Class II is 30.5 µM. However, this statistical difference in kinetic properties does not lead to a statistically significant difference in local performance or global tolerance (**Tables 5** **and** **6**). Therefore, the difference in kinetic properties is unable to explain the different clinical manifestations.

**Table 7:** Statistical comparison of the biochemical properties of the G6PD from the various classes.

Class			Kinetic Parameter (Medians)			
Reference	Comparison	Number of variants	G6PD activity (%)	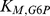 (µM)	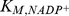 (µM)	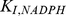 (µM)*
I		25	4.4	40.0	6.0	7.6
	II	12	*3.3* †	*42.2*	*5.4*	**30.5**
	III	21	**27.0** ‡	*39.0*	*5.2*	*18.0*
	IV	9	**77.5**	*48.0*	*6.3*	*24.0*
II		12	3.3	42.2	5.4	30.5
	III	21	**27.0**	*39.0*	*5.2*	*18.0*
	IV	9	**77.5**	*48.0*	*6.3*	*24.0*
III		21	27.0	39.0	5.2	18.0
	IV	9	*77.5*	*48.0*	*6.3*	*24.0*

The class in the first column (Reference) has its median kinetic properties compared to those of the classes in the second column (Comparison).

*Non-parametric methods of analysis were used in all cases except for the analysis of *K_I,NADPH_*, in which case parametric methods of analysis were available. For *K_I,NADPH_* the means are: 26.3, 60.9, 26.7 and 54.7 for Classes I through IV, respectively.

†Values italicized: p-value>0.05 (insignificant);

‡Values bold: p-value<0.05 (significant).

**Figure 4:**
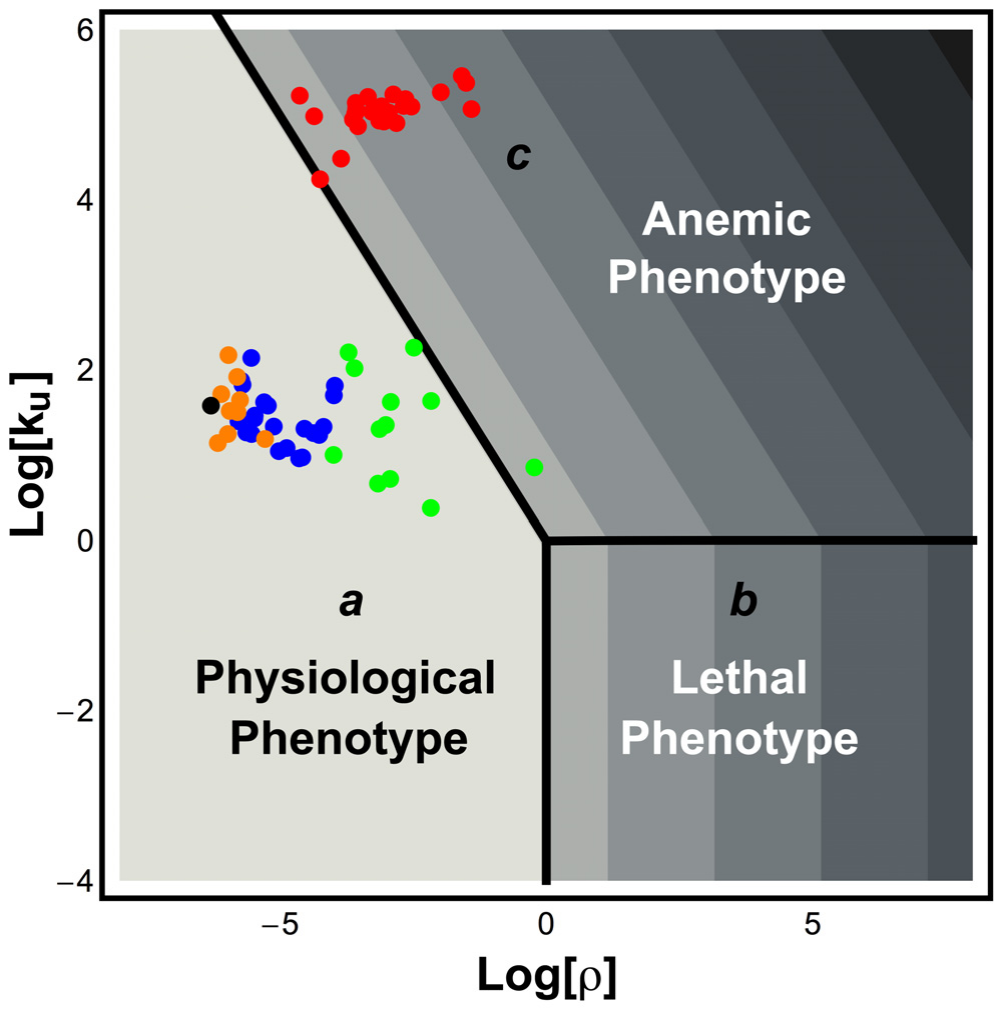
Location of operating points for Class I G6PD variants with an hypothetical increase in 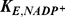. Class I variants of G6PD are subject to a different equilibrium between G6PD and NADP^+^ which results in increasing the value of 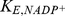 to 790 µM. Under this circumstance, because 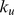, the y-axis, is directly proportional to 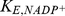, the operating points for Class I G6PD variants are located in Systemic Region ***c***. All other features of the design space are described in the legends of **Figures 2** and **3**.

Within the context of our model, there are several ways that these differences can be reconciled. A plausible example would be Class I variants having a higher value of 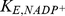. For instance, if the 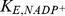 were 790 µM in Class I variants rather than the normal value of 7.9 µM, then the design space of the NADPH redox cycle would resemble **Figure 4**, and all Class I variants would operate in the poor region, Systemic Region ***c***, whereas most Class II variants would operate in the good region, Systemic Region ***a***.

### Infection by *Plasmodium falciparum*

It has been shown that *P. falciparum* can adapt to growth in G6PD-deficient erythrocytes by producing its own G6PD enzyme [34].

In one particular study [35], researchers purified G6PD from infected and uninfected erythrocytes. They found that infected erythrocytes had two different variants of G6PD: the G6PD that *P. falciparum* produces, responsible for 90% of the overall activity, and the host's G6PD, responsible for 10%.

**Figure 5:**
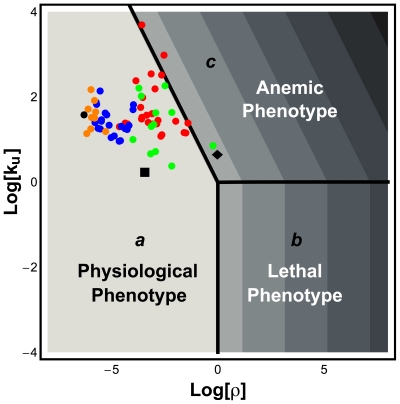
Location of uninfected (black diamond) and infected erythrocytes (black square) in design space. The operating point of the *P. falciparum*-infected erythrocyte is located in Systemic Region ***a*** whereas the operating point of uninfected erythrocytes is located in Systemic Region ***c***. All other features are described in the legends of **Figures 2** and **3**.

In **Table 8**, we list the experimental values for the kinetic properties of the host G6PD and the *P. falciparum* G6PD. By assuming normal values for the unknown independent variables and parameters, we are able to plot (**Figure 5**) the location of the operating point for the NADPH redox cycle in erythrocytes that are infected (black rectangle) or uninfected (black diamond) with *P. falciparum*.

**Table 8:** Kinetic properties of the *P. falciparum* G6PD and the host G6PD [35].

Kinetic Parameter	Host G6PD	*P. falciparum* G6PD
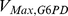 (µMs^−1^)*	0.2	4.8
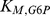 (µM)	27	11
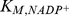 (µM)	1.4	0.8
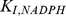 (µM)	16†	-

*The maximum velocity for synthesizing NADPH is twice the maximum velocity of G6PD (to take into account the GO6PD activity). For further details see [4];

†- from [9].

As can be seen in **Figure 5**, the uninfected erythrocytes endure a NADPH redox cycle with poor performance due to the G6PD deficiency (the operating point is located within Systemic Region ***c***). However, once *P. falciparum* infects an erythrocyte and synthesizes its own G6PD, the operating point of the NADPH redox cycle will move into the region of best performance (Systemic Region ***a***). Furthermore, we are able to assess the local performance and global tolerance of the NADPH redox cycle in human erythrocytes infected with *P. falciparum* (**Table 9**). The local performance is comparable to the performance of erythrocytes in which G6PD is of the B-form (**Table 4**). The major difference is in temporal responsiveness. Infected erythrocytes are more sluggish in responding to changes in GSSG. In terms of global tolerances, we observe that all tolerances for the infected erythrocyte are lower. This observation is correlated with the fact that the normal operating point of the NADPH redox cycle (G6PD B-form – black circle in **Figure 5**) is located further away from the boundary to Systemic Region ***c*** than the operating point of the infected erythrocytes (black rectangle). It is known that *P. falciparum*-infected erythrocytes are under a larger oxidative stress [36] and this effect may move its operating point in the design space further to the right (black rectangle).

**Table 9:** Evaluation of Local Performance and Global Tolerance for the NADPH redox cycle in human erythrocytes infected with *P. falciparum*.

Local Performance			
Criterion§	Preference|	Quantitative value†	Optimum value‡
*1*	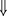	1.26	1
*2*	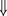	0.04	0
*3*	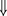	0.02	0
*4*	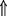	1.00	1
*5*	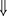	0.00	0
*6*	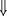	0.92	0
*7*	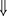	3.64	2

§The criteria were listed under the sub-section “Criteria for Functional Effectiveness” of the Methods Section;

|To improve performance, one must have either a high (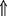) or a low (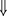) value for the associated criterion;

†The quantitative value was determined by using the data from **Tables 8** and **1** and the analytical expressions listed in **Table S1, S2** of the **Text S1**;

‡The optimum value was determined analytically except for criterion 7 which had to be determined numerically;

*[T represents tolerance to a fold decrease and T] represents tolerance to a fold increase.

We have to consider these results from the viewpoint of the *P. falciparum*. Upon infection of a G6PD-deficient erythrocyte, *P. falciparum* induces its own G6PD [34]. According to our analysis, this induction tends to correct the state of the redox cycle and *P. falciparum* can rely on a functional NADPH moiety supply unit. This scenario raises the question: is there a reason for *P. falciparum* to need a reliable NADPH moiety supply unit? *P. falciparum* digests the erythrocyte's hemoglobin and, as a consequence, produces 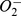, 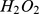 and OH radicals leading to a larger oxidative stress which the otherwise deficient cycle could not cope [36].

## Discussion

The piecewise power-law representation of the rate laws for G6PD and GSR allowed us to define the design space for the NADPH redox cycle. This design space has three phenotypic regions, each defined by a single type of stable steady state. However, the redox cycle only works properly in Systemic Region ***a***.

In comparing the local performance and global tolerance of the several possible cycles, it becomes apparent that an individual who has inherited the normal form of G6PD (B-form) displays one of the best possible NADPH redox cycles. One may argue that these functional advantages may explain the high prevalence of this form worldwide. In broader terms, we have demonstrated that there are statistically significant differences in local performance and global tolerance between cycles with a Class IV variant vs. a Class III/II/I variant. Individuals with a Class III variant of G6PD will experience a more sluggish cycle and an increased sensitivity to changes in glucose-6-phosphate and oxidized glutathione while, for example, not being able to tolerate an environment in which a large oxidative load would lead to a decrease in the concentration of glucose-6-phosphate to less than 0.3 µM. Individuals with a Class II G6PD variant will have a NADPH redox cycle that has an even slower response time as well as an even greater sensitivity to changes in the concentrations of NADP_total_, oxidized glutathione and glucose-6-phosphate. Furthermore, these patients will not tolerate an environment in which a large oxidative load would lead to a decrease in the concentration of glucose-6-phosphate to less than 2 µM.

### NADPH Redox Cycle Hypothesis

Individuals who have inherited a Class I variant of G6PD suffer from chronic hemolytic anemia, whereas individuals who have inherited a Class II variant develop significant hemolysis only when oxidative stress is induced. An increase in oxidative stress leads to a shift in the operating point toward higher values of ρ and, concomitantly, to a possible transition into Systemic Region *c*. These observations have prompted us to set forth the following hypothesis:

*When the NADPH redox cycle functions within Systemic Region* ***c*** *, the individual will suffer from hemolytic anemia. Furthermore, individuals with a Class II/III variant only transiently operate within Systemic Region* ***c*** *because they require an extra source of oxidative stress to develop clinical manifestations. However, individuals with a Class I variant always operate within Systemic Region* ***c*** *because their hemolytic anemia is chronic.*

To reconcile this hypothesis with our observations, we need to understand why Class I variants aren't mostly located within Systemic Region ***c*** (**Figure 3**). One possibility may reside in the stability of Class I variants of G6PD. *Cheng et al* [37] suggested that a correlation exists between mutations in evolutionary conserved amino acids and clinical manifestations. They showed the following trend: “as the severity of clinical consequences reduces from Class I, II and III, the percentage of mutations in the top pentad (conservation score >0.8) decreases from 51% (21/41) for class I to 41% (12/29) for class II and to 24% (5/21) for Class III.” Furthermore, the majority of Class I mutations are clustered near the dimer interface and NADP binding site for structural stability, or are deletion mutations [8]. In addition, of 40 Class I variants described in [38], 32 had highly decreased thermostability while 4 had decreased thermostability and 4 were classified as normal. Hence, one may speculate that Class I variants have a lower stability. In turn, a lower stability may lead to a sharp decrease in activity as erythrocytes age, eventually attaining a threshold (within Systemic Region ***c***) below which erythrocytes lyse. Chronic non-spherocytic haemolytic anemia ensues from the hematopoietic system being unable to compensate for the shortened erythrocyte lifespan with sufficiently increased reticulocytosis. On the other hand, because G6PD in these patients is sampled from a younger cell population, the overall G6PD activities are similar to those of Class II variants, which have longer erythrocyte lifespans.

A systematic reexamination of our modeling assumptions by means of the design space analysis suggests other testable possibilities for reconciling the observations with our hypothesis above. The most plausible among these is the prediction that Class I mutants exhibit a higher dissociation constant of the G6PD-NADP^+^ complex (**Figure 4**).

### Suggested Design Principle

Overall these results illustrate the importance of maintaining both good local performance and large global tolerance and suggest the following design principle: 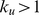, 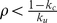 and 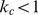. In order for Systemic Regime ***a*** to exist, 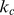 should be less than 1. In biological terms, this can be achieved by selecting for a GSR whose apparent Michaelis constant for NADPH is lower than the total concentration of NADP. To avoid Systemic Regime ***b***, which characterizes the worst local performance, 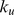 should be larger than 1. In this case, G6PD should display an apparent Michaelis constant for NADP^+^ than is higher than the total concentration of NADP. To achieve the best Systemic Region (***a***) 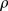 should be less than the ratio 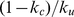. In biological terms, the apparent maximal velocity of GSR should be much lower than that for G6PD.

### Infection by *Plasmodium falciparum*

As one final point, it seems that natural selection has produced a parasite, *P. falciparum*, that is capable of adapting to G6PD-deficient erythrocytes by converting a deficient NADPH redox cycle into one that has both robust local behavior and large global tolerance. In the particular study that we have analyzed [35], the host inherited a G6PD variant that leads to an operating point in Systemic Regime ***c***. In order to adapt to this hostile environment, *P. falciparum* has to synthesize its own G6PD and is burdened with the associated energetic costs; the host cells, however, cannot benefit from this improved cycle because their ultimate fate is destruction by the *P. falciparum*.

### Conclusion

Our analysis of the NADPH redox cycle in human erythrocytes provides a clear example of how system design space concepts serve as a framework to understand quantitative physiological and pathological behavior. It allowed us to define and characterize three different phenotypic regions: physiological (Systemic regime ***a***), pathological (Systemic regime ***c***) and potentially lethal (Systemic regime ***b***). This example, and others [6], [7], [39], illustrates the utility of creating a design space that illuminates the function, design and fitness of the system. The system design space includes relationships among genotype, phenotype and environment. It provides an important framework to characterize the quantitative behavior of a system, and to compare wild-type and mutant variants. We know of no other approach that is able efficiently to accomplish these desirable objectives.

## Supporting Information

Text S1 Steady-state analysis: steady-state solutions and analysis of local performance.
(0.71 MB PDF)

Text S2 Annotated database of G6PD variants.
(0.55 MB PDF)
