# Supplementary material for: Relating Mutant Genotype to Phenotype via Quantitative Behavior of the NADPH Redox Cycle in Human Erythrocytes
Source: PLoS One. 2010 Sep 28;5(9):e13031. doi: 10.1371/journal.pone.0013031 (PMC2946920; doi:10.1371/journal.pone.0013031)
Supplement: Text S1 — Steady-state analysis: steady-state solutions and analysis of local performance. (0.71 MB PDF) [file pone.0013031.s001.pdf]

SUPPORTING TEXT S1

Steady-State Solutions

Based on the piecewise representation of the rate laws of G6PD and GSR in Log space (Eqns. 3 and 4 of the main text), we recognize that the NADPH redox cycle in human erythrocytes can operate under three different meaningful steady-state regimes and one unrealistic case with $u > k_u,\ c > k_c,\ \rho = 1$ (This case represents the system on a "knife edge".). In **Table S1-1**, we display the steady-state concentration of NADPH for each of the meaningful regimes, taking into account product-inhibition and the inhibition by 2,3-DPG of G6PD.

Table S1-1. Steady-state solutions of the NADPH redox cycle in human erythrocytes

Regime	$Log(v_{G6PD})$	$Log(v_{GSR})$	Steady-State Concentration of NADPH $NADPH_{steady-state}$
a	$Log\left(\frac{u}{k_u}\right)$	$Log(\rho)$	$\dfrac{NADP_{total} - \dfrac{\rho}{K_{M,G6P}+G6P}\left(K_{M,G6P}K_{E,NADP^+} + K_{M,NADP^+}G6P\left(1+\dfrac{2,3-DPG}{K_{I,2,3-DPG}}\right)\right)}{1+\dfrac{\rho\, G6P\, K_{M,NADP^+}}{K_{I,2,3-DPG}\left(K_{M,G6P}+G6P\right)}}$
b	$Log(1)$	$Log\left(\rho\frac{c}{k_c}\right)$	$NADP_{total}\dfrac{k_c}{\rho}$
c	$Log\left(\frac{u}{k_u}\right)$	$Log\left(\rho\frac{c}{k_c}\right)$	$\dfrac{-B+\sqrt{B^2+4\dfrac{\rho K_{M,NADP^+}G6P}{K^{App}_{M,NADPH}K_{I,NADPH}NADP_{total}\left(G6P+K_{M,G6P}\right)}}}{2\dfrac{\rho K_{M,NADP^+}G6P}{K^{App}_{M,NADPH}K_{I,NADPH}NADP_{total}\left(G6P+K_{M,G6P}\right)}}$ *

$$* \; B = \frac{1}{NADP_{total}} + \frac{\rho\left(K_{M,G6P}K_{E,NADP^+} + K_{M,NADP^+}G6P\left(1+\dfrac{2,3-DPG}{K_{I,2,3-DPG}}\right)\right)}{NADP_{total}K^{App}_{M,NADPH}\left(G6P+K_{M,G6P}\right)}$$

Analysis of Local Performance

Systemic Regime a

In **Table S1-2**, we show, for each criterion, the analytical expression that applies in Systemic Regime ***a***. It is apparent that a NADPH redox cycle operating in Systemic Regime ***a*** would be able to fulfill all of the criteria that we have defined.

Table S1-2. Evaluation of the local performance in Systemic Regime ***a***.

Criterion	Preference‖	Capable of being fulfilled	Analytical Expression	Optimum Value
1	⇓	+	$\sum_{i=1}^{10}\left\lvert S\left(NADPH_{steady-state}, p_i\right)\right\rvert$ $p_i = \left\{\begin{matrix} K_{I,2,3-DPG}, K_{M,GSSG}, K_{M,NADP^+}, K_{I,NADPH}, K_{M,G6P}, \\ K_{E,NADP^+}, V_{Max,G6PD}, V_{Max,GSR}, NADP_{total}, 2,3-DPG \end{matrix}\right\}$ *	1
2	⇓	+	$\frac{GSSG\, K_{M,GSSG}\, K_{I,NADPH} V_{Max,G6PD} V_{Max,GSR}\, B}{G6P\, K_{I,2,3-DPG} NADPH_{steady-state} A^2}$ †	0
3	⇓	+	$\frac{K_{M,G6P} K_{E,NADP^+} K_{I,NADPH} V_{Max,GSR}\, GSSG}{G6P\, K_{I,2,3-DPG} NADPH_{steady-state} A}$	0
4	⇑	+	$\frac{K_{M,GSSG}}{GSSG + K_{M,GSSG}}$	1
5	⇓	+	$\frac{GSSG}{K_{M,GSSG} + GSSG}$	0
6	⇓	+	$\left\lvert\frac{1}{\lambda_a}\right\rvert = \frac{K_{I,NADPH} V_{Max,G6PD}\left(GSSG + K_{M,GSSG}\right)^2 B}{G6P\, K_{I,2,3-DPG} A^2}$	0
7	⇓	+	$\sum_{i=1}^{12}\left\lvert S(\lambda_a, p_i)\right\rvert$ $p_i = \left\{\begin{matrix} GSSG, K_{M,GSSG}, G6P, K_{M,G6P}, K_{M,NADP^+}, K_{E,NADP^+}, K_{I,NADPH}, \\ 2,3-DPG, K_{I,2,3-DPG}, NADP_{total}, V_{Max,G6PD}, V_{Max,GSR} \end{matrix}\right\}$ *	2‡

‖ To improve performance, one must have either a high (⇑) or a low (⇓) value for the associated criterion.

* These analytical expressions are too cumbersome to display. Therefore, sufficient information is presented to reproduce the results.

† $A = \left(GSSG + K_{M,GSSG}\right) K_{I,NADPH} V_{Max,G6PD} + K_{M,NADP^+} V_{Max,GSR} GSSG$

$B = K_{M,G6P} K_{E,NADP^+} K_{I,NADPH} K_{I,2,3-DPG} + K_{M,NADP^+} G6P\left(K_{I,NADPH}\, 2,3-DPG + K_{I,2,3-DPG}\left(NADP_{total} + K_{I,NADPH}\right)\right)$

‡ The optimum value was determined by numerical simulation.

Systemic Regime b

In **Table S1-3**, we show, for each criterion, the analytical expression that applies in Systemic Regime ***b***. Contrary to the results for Systemic Regime ***a***, the local performance in Systemic Regime ***b*** cannot fulfill criteria 4 and 5. This indicates that the supply of NADPH (V_{G6PD}) would not respond to changes in the concentration of GSSG, which would prevent the cycle from performing its function. Furthermore, as can be seen from **Table S1-3**, the response time only depends on $V_{Max,GSR}$ and $K_{M,NADPH}$. It, however, does not depend on the concentration of GSSG. Therefore, the importance of this responsiveness becomes questionable.

Table S1-3. Evaluation of the local performance in Systemic Regime ***b***.

**Criterion**	**Preference**||	**Capable of being fulfilled**	**Analytical Expression**	**Optimum Value**
1	⇓	+	$3+\frac{K_{M,G6P}}{K_{M,G6P}+G6P}$	3
2	⇓	+	0	0
3	⇓	+	$\frac{K_{M,G6P}}{K_{M,G6P}+G6P}$	0
4	⇑	-	0	1
5	⇓	-	NA†	NA
6	⇓	+	$\frac{K_{M,NADPH}}{V_{Max,GSR}}$	0
7	⇓	+	2	2

|| To improve performance, one must have either a high (⇑) or a low (⇓) value for the associated criterion.

† NA – Not applicable

Systemic Regime c

In **Table S1-4**, we show, for each criterion, the analytical expression that applies in Systemic Regime ***c***. As in Systemic Regime ***b***, criteria 4 and 5 cannot be fulfilled in Systemic Regime ***c***. In addition, even though Systemic Regime ***c*** can have a fast response time (Criterion 6), it will not be with respect to changes in the concentration of GSSG. Therefore, as in Systemic Regime ***b***, the importance of this responsiveness becomes questionable.

Table S1-4. Evaluation of the local performance in Systemic Regime ***c***.

Criterion	Preference‖	Capable of being fulfilled	Analytical Expression	Optimum Value
1	⇓	+	$\sum_{i=1}^{10} \left\| S(NADPH_{steady-state}, p_i) \right\|$ $p_i = \begin{Bmatrix} K_{M,NADPH}, K_{M,G6P}, K_{E,NADP^+}, K_{I,NADPH}, K_{I,2,3-DPG}, \\ K_{M,NADP^+}, 2,3-DPG, NADP_{total}, V_{Max,G6PD}, V_{Max,GSR} \end{Bmatrix}$ *	1§
2	⇓	+	0	0
3	⇓	+	$\frac{K_{M,G6P}K_{E,NADP^+}K_{I,NADPH}K_{I,2,3-DPG}V_{Max,GSR}}{\sqrt{K_{I,NADPH}\begin{pmatrix} 4G6P^2K_{I,2,3-DPG}^2K_{M,NADPH}K_{M,NADP^+}NADP_{total}V_{Max,G6PD}V_{Max,GSR} \\ +K_{I,NADPH}C^2 \end{pmatrix}}}$ ‡	0
4	⇑	-	0	1
5	⇓	-	NA†	NA
6	⇓	+	$\left\| \frac{1}{\lambda_c} \right\|$ *	0§
7	⇓	+	$\sum_{i=1}^{12} \left\| S(\lambda_c, p_i) \right\|$ $p_i = \begin{Bmatrix} K_{M,NADPH}, K_{M,G6P}, K_{E,NADP^+}, K_{I,NADPH}, K_{I,2,3-DPG}, K_{M,NADP^+}, \\ V_{Max,G6PD}, V_{Max,GSR}, 2,3-DPG, NADP_{total}, G6P \end{Bmatrix}$ *	2§

‖ To improve performance, one must have either a high (⇑) or a low (⇓) value for the associated criterion.

* These analytical expressions are too cumbersome to display. Therefore, sufficient information is presented to reproduce the results.

† NA – Not applicable

‡ $C = K_{M,NADPH}K_{I,2,3-DPG}V_{Max,G6PD}G6P + K_{M,G6P}K_{E,NADP^+}K_{I,2,3-DPG}V_{Max,GSR} + K_{M,NADP^+}V_{Max,GSR}G6P\left(2,3-DPG + K_{I,2,3-DPG}\right)$

§ The optimum value was determined by numerical simulation.

The optimum value of Criterion *1* in Systemic Regime ***c*** is 1, whereas that in Systemic Regime ***b*** is 3. Therefore, since Systemic Regimes ***b*** and ***c*** share the same optimum values for the remaining criteria, we conclude that overall local performance in Systemic Regime ***c*** is better than that in Systemic Regime ***b***.
