# Supplementary material for: Relating Mutant Genotype to Phenotype via Quantitative Behavior of the NADPH Redox Cycle in Human Erythrocytes
Source: PLoS One. 2010 Sep 28;5(9):e13031. doi: 10.1371/journal.pone.0013031 (PMC2946920; doi:10.1371/journal.pone.0013031)
Supplement: Text S2 — Annotated database of G6PD variants. (0.55 MB PDF) [file pone.0013031.s002.pdf]

SUPPORTING TEXT S2

Variants of G6PD

More than 400 putative variants of the G6PD enzyme in humans have been identified. In addition, the biochemical characterization of partially purified mutants of G6PD enzymes has revealed even further heterogeneity at the level of the kinetic parameters, heat stability, pH optimum and the utilization of substrate analogues. The results of such measurements are, of course, dependent on the methodology used. However, in 1967, a committee of the World Health Organization (WHO) recommended standard techniques for the biochemical characterization of G6PD variants [1]. As of June 1989, nearly 300 putative variants had been characterized by these standard methods, had been regarded as unique, and had been given a name based on its geographical localization. An additional 100 variants were characterized by methods that differ from those recommended by the WHO. The properties of the putative variants described up to June 1989 were summarized in [2]. However, of these putative variants only 160 mutations in the *G6PD* gene have actually been characterized [3].

In order to minimize the number of assumptions in our model (described in the main text), we considered only variants that were characterized according to the WHO guidelines and had experimental values for all the kinetic parameters of the enzyme: $K_{M,G6P}$, $K_{M,NADP^+}$, $K_{I,NADPH}$ and $V_{Max,G6PD}$. Of the nearly 300 variants described in [2], only 96 variants fit these criteria. In checking the data in [2] against the original papers, we found several discrepancies.

These included:

a) Minor inaccuracies between the numerical values reported in [2] and the data in the original papers (less than 20% error);

b) Major inaccuracies between the numerical values reported in [2] and the data in the original papers (more than 20% error);

c) The original paper placed the variant in one class, whereas [2] included it in a different (wrong) class;

d) The original paper misclassified a variant and [2] included it in the correct class.

Furthermore, of the 96 variants under consideration we had to exclude 29 due to the following reasons:

e) The biochemical characterization of 18 G6PD variants was unpublished. Given the number of errors and misclassifications of variants that we encountered in [2], we believe that it would be more conservative to excluded these variants from our analysis;

f) The original literature states that the activity of G6PD in the red blood cell is undetectable for 5 G6PD variants;

g) We were unable to document $K_{I,NADPH}$ values for 3 G6PD variants in the original literature, even though [2] states values for them;

h) The original literature involved cases with complications that confound the link between the G6PD deficiency and the clinical manifestations for 2 particular variants of G6PD. Therefore, proper classification of the mutant variant is questionable;

i) One G6PD variant was found to be genetically identical to a previously described G6PD variant;

In the following tables, we provide the kinetic information for the 67 G6PD variants that we included in our analysis. Except for any irregularity that we found in the original literature, the discrepancies between [2] and the original literature were resolved in favor of the data documented in the original literature.

Table S2-1. Properties of Class I variants of G6PD for which there are, according to [2], numerical values for all four of the following parameters: G6PD activity, $K_{M,G6P}$, $K_{M,NADP^+}$ and $K_{I,NADPH}$

Name	Included in the model	Problem†	Reference	Kinetic Parameter			
				G6PD activity (% of normal)	$K_{M,G6P}$ (μM)	$K_{M,NADP^+}$ (μM)	$K_{I,NADPH}$ (μM)
Ogikubo	Y		[4]	3	47	3	11.5
Yokohama	Y		[4]	1.9	70	6.1	2.9
Atlanta	Y		[5]	25	63	4.5	5.5
Nagano	Y	**a**	[6]	5.5	28	9.1	3.1
Lincoln Park	Y	**a**	[7]	6.5	32.9	8.8	21.8
West Town	Y	**a**	[7]	6.7	59.3	8.2	21.6
Guadalajara	Y	**a**	[8]	14	36	5.3	22
Tokushima	Y	**a**	[9]	3	50	27	7.1
Tokyo	Y	**a**	[9]	4.4	65	5.5	7.1
Aarau	Y	**a**	[10]	6.67	37.9	6.5	290
Kanazawa	Y	**a**	[11]	7	43	6	5.2
Regensburg	Y	**a**	[12]	6	14	15	40
Walter Reed	Y	**a**	[13]	5	40	5.4	12.9
Sendagi	Y	**a**	[14]	8.4	11.2	4.4	15.3
Iwate	Y	**a**	[15]	2.3	37	40	3
Moosburg	Y	**a**	[16]	3.6	32	5.2	100
Wayne	Y	**b**	[17]	6	30.9	78.2	20.3
Tsukui	Y	**b**	[18]	1.5	100	4	12.6
Kurume	Y	**c**	[19]	0.8	43	5.7	1.9
Fukushima	Y	**c**	[19]	2.8	31	5	4.4
Wakayama	Y	**c**	[19]	4.5	46	6.5	3.2
Yamaguchi	Y	**c**	[19]	3.5	37	15.2	7.6
Asahikawa	Y	**c, a**	[20]	3.8	29.9	18.3	2.1
Gifu	Y	**c, b**	[21]	2.9	48	3.1	7.1
Velletri	Y	**c, b**	[22]	2.1	140	4.4	30
Birmingham	N	**h, a**	[23]	<5	12	22	10.91
Hotel Dieu	N	**f**	[24]	-	26	11.4	30
San Francisco	N	**f**	[25]	-	77	8.4	2.7
Akita	N	**f**	[4]	-	33	3.3	7.9
Dothan	N	**f, a**	[26]	-	60	5.25	5.6
Linda Vista	N	**e**		<.13	3	7	7.1
Hawaii	N	**e**		23	39	9.7	28.6
Iowa City	N	**e**		1.3	48	6.9	11.6
Springfield	N	**e**		11	33	4	4
Indianapolis	N	**e**		5	56.7	5.6	21.2
Gastonia	N	**e**		4	66	7	18
Pompton Plains	N	**e**		0.5	10.8	4	25.7
Iowa	N	**e**		12.5	65	4.4	15.8
Loma Linda	N	**e**		0.8	69.6	7.9	9.5
Santa Barbara	N	**e**		6.5	51.3	10.6	32.2

† **a**, Minor inaccuracies; **b** Major inaccuracies; **c**, Wrong classification of the variant in [2]; **e**, Unpublished data; **h**, Conflicting information about proper classification; **f**, Undetectable G6PD enzyme activity; **g**, Undocumented value for $K_{I,NADPH}$.

Table S2-2. Properties of Class II variants of G6PD for which there are, according to [2], numerical values for all four of the following parameters: G6PD activity, $K_{M,G6P}$, $K_{M,NADP^+}$ and $K_{I,NADPH}$

Name	Included in the model	Problem†	Reference	Kinetic Parameter			
				G6PD activity (% of normal)	$K_{M,G6P}$ (μM)	$K_{M,NADP^+}$ (μM)	$K_{I,NADPH}$ (μM)
Ankara	Y		[27]	8	52	15	42
Bielefeld	Y		[28]	2.7	21.7	7	170
Nukus	Y		[29]	0.5	127.4	0.3	45
Viangchan	Y		[30]	3	105	12	19
Aachen	Y		[31]	3	65	22.5	7
Avenches	Y	**a**	[16]	9.1	30.9	2.8	180
Cagliari	Y	**a**	[32]	5.1	54	4.8	10
Mediterranean	Y	**a**	[33], [34]	3.5	23	1.4	16
Iserlohn	Y	**b**	[12]	6	22	14	105
Menorca	Y	**b**	[35]	2.4	14.5	1.9	118
Fukuoka	Y	**b, c**	[21]	6.4	92	6	7.9
Tashkent	Y	**d**	[29]	1.5	32.3	0.2	11.2
Union	N	**g, b**	[36]	2.5	10	4.4	-
San Jose	N	**f, g, b**	[37], [38]	-	50	5	-
Pawnee	N	**e**		6.8	50	20	18.6
Laos	N	**e**		1.4	68.1	7.2	115.6
Waterloo	N	**e**		5.8	19.7	6.9	4
Dallas	N	**e**		<.1	13.8	2.9	24.2

† **a**, Minor inaccuracies; **b** Major inaccuracies; **c**, Wrong classification of the variant in [2]; **d**, Wrong classification in the original reference but correct in [2]; **e**, Unpublished data; **f**, Undetectable G6PD enzyme activity; **g**, Undocumented value for $K_{I,NADPH}$.

Table S2-3. Properties of Class III variants of G6PD for which there are, according to [2], numerical values for all four of the following parameters: G6PD activity, $K_{M,G6P}$, $K_{M,NADP^+}$ and $K_{I,NADPH}$

Name	Included in the model	Problem†	Reference	Kinetic Parameter			
				G6PD activity (% of normal)	$K_{M,G6P}$ (μM)	$K_{M,NADP^+}$ (μM)	$K_{I,NADPH}$ (μM)
Castilla	Y		[39]	20	50	4.5	6.8
Metaponto	Y		[34]	27	47	3	12
Chiapas	Y		[40]	15	25	2.6	23
Gallura	Y		[41]	15	40	4.5	72
Agrigento	Y		[41]	25	30	3	40
Vientiane	Y		[42]	56	39	10.5	85
Central City	Y	**a**	[43]	14	43	3.6	4.6
Alabama	Y	**a**	[44]	14	71	11.7	6.8
Tepic	Y	**a**	[38]	11.3	48.2	9.2	20.1
Nagasaki-2	Y	**a**	[45]	52	32	10.2	30
Hiroshima-1	Y	**a**	[45]	52	39	4.5	47
Hiroshima-3	Y	**a**	[45]	33	35	7.1	31
Ube	Y	**a**	[46]	36.6	52.5	5.4	48.8
Konan	Y	**a**	[47]	44.9	39.6	5.7	9.3
Kamiube	Y	**a**	[47]	46	37	3.9	7.9
Jalisco	Y	**a**	[48]	55.7	32.9	5.2	15.8
Los Angeles	Y	**a**	[49]	34	17	11	9.3
Musashino	Y	**a**	[50]	17.5	31.5	2.3	8.3
A^-	Y	**b**	[51], [38]	14	50	5	13
Nagasaki-3	Y	**b**	[45]	63	71	7	51
Gabrovizza	Y	**b**	[52]	24	11.2	6	18
Matera	N	**i, a**	[34]	16.5	47	2	13
Hofu	N	**g**	[53]	12.1	25	5	-
Great Lakes	N	**e**		11.5	61.4	9.1	4
Mercury	N	**e**		25.2	8.1	7.1	27.4
Fort Pierce	N	**e**		31.2	54.7	7.9	5.4

† **a**, Minor inaccuracies; **b** Major inaccuracies; **e**, Unpublished data; **g**, Undocumented value for $K_{I,NADPH}$; **i**, genetically identical to A^-

Table S2-4. Properties of Class IV variants of G6PD for which there are, according to [2], numerical values for all four of the following parameters: G6PD activity, $K_{M,G6P}$, $K_{M,NADP^+}$ and $K_{I,NADPH}$

Name	Included in the model	Problem†	Reference	Kinetic Parameter			
				G6PD activity (% of normal)	$K_{M,G6P}$ (µM)	$K_{M,NADP^+}$ (µM)	$K_{I,NADPH}$ (µM)
Laguna	Y		[54]	64	20.7	7.6	43
PinarDelRio	Y		[55]	100	48.5	3.7	225
Kiwa	Y		[47]	64	42	6.1	8.6
Morelia	Y	**a**	[48]	77.5	44.18	15.31	8.55
Titusville	Y	**a**	[56]	69	48	12.1	16
Cuiaba	Y	**a**	[57]	83	138	4.8	120
A	Y	**a**	[34], [58]	84	50	4	7
Nagasaki-1	Y	**a**	[45]	79	66	9.6	24
Regar	Y	**a**	[59]	62.3	29.3	6.3	40
Huntsville	N	**h, a**	[60]	86	43	3	6.25
Lynn	N	**e**		69	54	8.3	6.5

† **a**, Minor inaccuracies; **e**, Unpublished data; **h**, Conflicting information about proper classification.

REFERENCES

1. Betke K, Beutler E, Brewer GJ, Kirkman HN, Luzzatto L, et al. (1967) Standardization of Procedures for the study of glucose 6-phosphate dehydrogenase. Report of a WHO scientific group. World Health Organ Tech Rep Ser 366.
2. Beutler E (1990) The Genetics of Glucose-6-Phosphate Dehydrogenase Deficiency. Seminars in Hematology 27: 137-164.
3. Mason PJ, Bautista JM, Gilsanz F (2007) G6PD deficiency: the genotype-phenotype association. Blood Rev 21: 267-283.

4. Miwa S, Fujii H, Nakashima K, Miura Y, Yamada K, et al. (1978) Three new electrophoretically normal glucose-6-phosphate dehydrogenase variants associated with congenital nonspherocytic hemolytic anemia found in Japan: G6PD Ogikubo, Yokohama, and Akita. Hum Genet 45: 11-17.

5. Beutler E, Keller JW, Matsumoto F (1976) A new glucose-6-p dehydrogenase (G-6-PD) variant associated with nonspherocytic hemolytic anemia: G-6-PD Atlanta. IRCS Medical Science 4: 579.

6. Takahashi K, Fujii H, Takegawa S, Tani K, Hirono A, et al. (1982) A New Glucose-6-Phosphate-Dehydrogenase Variant (G6pd Nagano) Associated with Congenital Hemolytic-Anemia. Human Genetics 62: 368-370.

7. Honig GR, Habacon E, Vida LN, Matsumoto F, Beutler E (1979) Three new variants of glucose-6-phosphate dehydrogenase associated with chronic nonspherocytic hemolytic anemia: G-6-PD Lincoln Park, G-6-PD Arlington Heights, and G-6-PD West Town. Am J Hematol 6: 353-360.

8. Vaca G, Ibarra B, Romero F, Olivares N, Cantu JM, et al. (1982) G-6-PD Guadalajara. A new mutant associated with chronic nonspherocytic hemolytic anemia. Hum Genet 61: 175-176.

9. Miwa S, Ono J, Nakashima K, Abe S, Kageoka T (1976) Two new glucose 6-phosphate dehydrogenase variants associated with congenital nonspherocytic hemolytic anemia found in Japan: GD(-) Tokushima and GD(-) Tokyo. Am J Hematol 1: 433-442.

10. Gahr M, Schroter W, Sturzenegger M, Bornhalm D, Marti HR (1976) Glucose-6-phosphate dehydrogenase (G-6-PD) deficiency in Switzerland. Demonstration of a new variant (G-6-PD Aarau) with chronic nonsphaerocytic haemolytic anaemia. Helv Paediatr Acta 31: 159-166.

11. Kitao T, Ito K, Hattori K, Matsuki T, Yoneyama Y (1982) G6Pd Kanazawa: a new variant of glucose-6-phosphate dehydrogenase associated with congenital nonspherocytic hemolytic anemia. Acta Haematol 68: 131-135.

12. Eber SW, Gahr M, Schroter W (1985) Glucose-6-phosphate dehydrogenase (G6PD) Iserlohn and G6PD Regensburg: two new severe enzyme defects in German families. Blut 51: 109-115.

13. Beutler E, Hartman K, Gelbart T, Forman L (1986) G-6-PD Walter Reed: possible insight into "structural" NADP in G-6-PD. Am J Hematol 23: 25-30.

14. Morisaki T, Fujii H, Takegawa S, Tani K, Hirono A, et al. (1983) G6PD Sendagi: a new glucose-6-phosphate dehydrogenase variant associated with congenital hemolytic anemia. Hum Genet 65: 214-215.

15. Kanno H, Takano T, Fujii H, Tani K, Morisaki T, et al. (1988) A new glucose-6-phosphate dehydrogenase variant (G6PD Iwate) associated with congenital non-spherocytic hemolytic anemia. Nippon Ketsueki Gakkai Zasshi 51: 715-719.

16. Pekrun A, Eber SW, Schroter W (1989) G6PD Avenches and G6PD Moosburg: biochemical and erythrocyte membrane characterization. Blut 58: 11-14.

17. Ravindranath Y, Beutler E (1987) Two new variants of glucose-6-phosphate dehydrogenase associated with hereditary non-spherocytic hemolytic anemia: G6PD Wayne and G6PD Huron. Am J Hematol 24: 357-363.

18. Ogura H, Morisaki T, Tani K, Kanno H, Tsutsumi H, et al. (1988) A new glucose-6-phosphate dehydrogenase variant (G6PD Tsukui) associated with congenital hemolytic anemia. Hum Genet 78: 369-371.

19. Miwa S, Fujii H, Nakatsuji T, Ishida Y, Oda E, et al. (1978) Four new electrophoretically slow-moving glucose 6-phosphate dehydrogenase variants associated with congenital nonspherocytic hemolytic anemia found in Japan: Gd(-) Kurume, Gd(-) Fukushima, Gd(-) Yamaguchi and Gd(-) Wakayama. Am J Hematol 5: 131-138.

20. Takizawa T, Fujii H, Takegawa S, Takahashi K, Hirono A, et al. (1984) A unique electrophoretic slow-moving glucose 6-phosphate dehydrogenase variant (G6PD Asahikawa) with a markedly acidic pH optimum. Hum Genet 68: 70-72.

21. Fujii H, Miwa S, Takegawa S, Takahashi K, Hirono A, et al. (1984) Gd(-) Gifu and Gd(-) Fukuoka. Two new variants of glucose-6-phosphate dehydrogenase found in Japan. Hum Genet 66: 276-278.

22. Mandelli F, Amadori S, Delaurenzi A, Kahn A, Isacchi G, et al. (1977) Glucose-6-Phosphate-Dehydrogenase Velletri - New Variant with Reduced Activity in a Patient with Congenital Non-Spherocytic Hemolytic-Anemia. Acta Haematologica 57: 121-126.

23. Prchal JT, Crist WM, Malluh A, Vitek A, Tauxe WN, et al. (1980) A new glucose-6-phosphate dehydrogenase deficient variant in a patient with Chediak-Higashi syndrome. Blood 56: 476-480.

24. Kahn A, Dao C, Cottreau D, Bilski-Pasquier G (1977) 'Gd(-) Hotel Dieu': a new G-6PD variant with chronic hemolysis in a Negro patient from Senegal. Hum Genet 39: 353-357.

25. Mentzer WC, Jr., Warner R, Addiego J, Smith B, Walter T (1980) G6PD San Francisco: a new variant of glucose-6-phosphate dehydrogenase associated with congenital nonspherocytic hemolytic anemia. Blood 55: 195-198.

26. Prchal J, Moreno H, Conrad M, Vitek A (1979) G-6-PD Dothan: A new variant associated with chronic hemolytic anemia. IRCS Medical Science 7: 348.

27. Kahn A, North ML, Messer J, Boivin P (1975) G-6PD "ankara". a new G-6PD variant with deficiency found in a Turkish family. Humangenetik 27: 247-250.

28. Gahr M, Bornhalm D, Schroter W (1977) [Biochemical characterization of a new variant of glucose-6-phosphate dehydrogenase (G-6-PD) deficiency with favism: G-6-PD Bielefeld (author's transl)]. Klin Wochenschr 55: 379-384.

29. Yermakov N, Tokarev J, Chernjak N, Schonian G, Grieger M, et al. (1981) New stable mutant (Gd(-) variants: G6PD Tashkent and G6PD Nucus. Molecular basis of hereditary enzyme deficiency. Acta Biol Med Ger 40: 559-562.

30. Poon MC, Hall K, Scott CW, Prchal JT (1988) G6PD Viangchan: a new glucose 6-phosphate dehydrogenase variant from Laos. Hum Genet 78: 98-99.

31. Kahn A, Esters A, Habedank M (1976) Gd(-) Aachen, a New Variant of Deficient Glucose-6-Phosphate-Dehydrogenase - Clinical, Genetic, and Biochemical Aspects. Human Genetics 32: 171-180.

32. Morelli A, Benatti U, Guida L, De Flora A (1984) G6PD Cagliari: a new low activity glucose 6-phosphate dehydrogenase variant characterized by enhanced intracellular lability. Hum Genet 66: 62-65.

33. Kirkman HN, Schettin.F, Pickard BM (1964) Mediterranean Variant of Glucose-6-Phosphate Dehydrogenase. Journal of Laboratory and Clinical Medicine 63: 726-&.

34. Vulliamy TJ, Durso M, Battistuzzi G, Estrada M, Foulkes NS, et al. (1988) Diverse Point Mutations in the Human Glucose-6-Phosphate-Dehydrogenase Gene Cause Enzyme Deficiency and Mild or Severe Hemolytic-Anemia. Proceedings of the National Academy of Sciences of the United States of America 85: 5171-5175.

35. Vives Corrons JL, Pujades A (1982) Heterogeneity of "Mediterranean type" glucose-6-phosphate dehydrogenase (G6PD) deficiency in Spain and description of two new variants associated with favism. Hum Genet 60: 216-221.

36. Yoshida A, Baur EW, Moutlsky AG (1970) A Philippino glucose-6-phosphate dehydrogenase variant (G6PD Union) with enzyme deficiency and altered substrate specificity. Blood 35: 506-513.

37. Castro GA, Snyder LM (1974) G6PD San Jose: a new variant characterized by NADPH inhibition studies. Humangenetik 21: 361-363.

38. Lisker R, Perez-Briceno R, Beutler E (1985) A new glucose-6-phosphate dehydrogenase variant, Gd(-) Tepic, characterized by moderate enzyme deficiency and mild episodes of hemolytic anemia. Hum Genet 69: 19-21.

39. Lisker R, Briceno RP, Zavala C, Navarrette JI, Wessels M, et al. (1977) A glucose 6-phosphate dehydrogenase Gd (-) Castilla variant characterized by mild deficiency associated with drug-induced hemolytic anemia. J Lab Clin Med 90: 754-759.

40. Lisker R, Perezbriceno R, Agrilar L, Yoshida A (1978) Variant Glucose-6-Phosphate-Dehydrogenase Gd(-) Chiapas Associated with Moderate Enzyme Deficiency and Occasional Hemolytic-Anemia. Human Genetics 43: 81-84.

41. Sansone G, Perroni L, Yoshida A (1975) Glucose-6-Phosphate-Dehydrogenase Variants from Italian Subjects Associated with Severe Neonatal Jaundice. British Journal of Haematology 31: 159-165.

42. Kahn A, North ML, Cottreau D, Giron G, Lang JM, et al. (1978) G6pd Vientiane - New Glucose-6-Phosphate-Dehydrogenase Variant with Increased Stability. Human Genetics 43: 85-89.

43. Csepreghy M, Yeilding A, Lilly M, Hall K, Scott CW, et al. (1988) Characterization of a new glucose-6-phosphate dehydrogenase variant: G6PD Central City. Am J Hematol 28: 61-62.

44. Prchal JT, Hall K, Csepreghy M, Lilly M, Berkow R, et al. (1988) Two apparent glucose-6-phosphate dehydrogenase variants in normal XY males: G6PD Alabama. Am J Med 84: 517-523.

45. Kageoka T, Satoh C, Goriki K, Fujita M, Neriishi S, et al. (1985) Electrophoretic Variants of Blood Proteins in Japanese .4. Prevalence and Enzymologic Characteristics of Glucose-6-Phosphate-Dehydrogenase Variants in Hiroshima and Nagasaki. Human Genetics 70: 101-108.

46. Nakashima K, Ono J, Abe S, Miwa S, Yoshida A (1977) G6pd Ube, a Glucose-6-Phosphate-Dehydrogenase Variant Found in 4 Unrelated Japanese Families. American Journal of Human Genetics 29: 24-30.

47. Nakatsuji T, Miwa S (1979) Incidence and Characteristics of Glucose-6-Phosphate-Dehydrogenase Variants in Japan. Human Genetics 51: 297-305.

48. Vaca G, Ibarra B, Cruz DG, Medina C, Romero F, et al. (1985) G-6-Pd Jalisco and G-6-Pd Morelia - 2 New Mexican Variants. Human Genetics 71: 82-85.

49. Beutler E, Matsumoto F (1977) A new Glucose 6 Phosphate Dehydrogenase Variant: Glucose 6 phosphate Dehydrogenase Los Angeles. IRCS (International Research Communications System) Medical Science Library Compendium 5: 89.

50. Kumakawa T, Suzuki S, Fujii H, Miwa S (1987) Frequency of Glucose-6-Phosphate-Dehydrogenase (G6pd) Deficiency in Tokyo and a New Variant - G6pd Musashino. Acta Haematologica Japonica 50: 25-28.

51. Yoshida A, Stamatoyannopoulos G, Motulsky AG (1967) Negro variant of glucose-6-phosphate dehydrogenase deficiency (A-) in man. Science 155: 97-99.

52. Ventura A, Panizon F, Soranzo MR, Veneziano G, Sansone G, et al. (1984) Congenital Dyserythropoietic Anemia Type-Ii Associated with a New Type of G6pd-Deficiency (G6pd-Gabrovizza). Acta Haematologica 71: 227-234.

53. Miwa S, Nakashima K, Ono J, Fujii H, Suzuki E (1977) 3 Glucose-6-Phosphate Dehydrogenase Variants Found in Japan. Human Genetics 36: 327-334.

54. Weimer TA, Schuler L, Beutler E, Salzano FM (1984) Gd (+) Laguna, a New Rare Glucose-6-Phosphate-Dehydrogenase Variant from Brazil. Human Genetics 65: 402-404.

55. Gonzalez R, Wade M, Estrada M, Svarch E, Colombo B (1977) G6pd Pinar Del-Rio - New Variant Discovered in a Cuban Family. Biochemical Genetics 15: 909-913.

56. Csepreghy M, Hall MK, Berkow RL, Jackson S, Prchal JT (1989) Characterization of a New G6pd Variant - G6pd Titusville. American Journal of the Medical Sciences 297: 114-117.

57. Barretto OCD, Nonoyama K (1987) Gd(+)Cuiaba, a New Rare Glucose-6-Phosphate-Dehydrogenase Variant Presenting Normal Activity. Human Genetics 77: 201-202.

58. Boyer SH, Porter IH, Weilbach.Rg (1962) Electrophoretic Heterogeneity of Glucose-6-Phosphate Dehydrogenase and Its Relationship to Enzyme Deficiency in Man. Proceedings of the National Academy of Sciences of the United States of America 48: 1868-&.

59. Ermakov NV, Chernyak NB, Tokarev YN (1983) Properties of New Variant of Glucose-6-Phosphate-Dehydrogenase (Regar Variant) - Glucose-Metabolism in Erythrocytes Containing Abnormal Enzyme. Biochemistry-Moscow 48: 500-506.

60. Hall K, Schreeder MT, Prchal JT (1988) G6pd Huntsville - a New Glucose-6-Phosphate-Dehydrogenase Associated with Chronic Hemolytic-Anemia. Human Genetics 79: 90-91.
